# Labeling Strategies for Surface-Exposed Protein Visualization and Determination in *Plasmodium falciparum* Malaria

**DOI:** 10.3389/fcimb.2022.914297

**Published:** 2022-06-10

**Authors:** Jinfeng Shao

**Affiliations:** Laboratory of Malaria and Vector Research, National Institute of Allergy and Infectious Diseases, National Institutes of Health, Rockville, MD, United States

**Keywords:** surface-exposed protein, malaria, *Plasmodium falciparum*, protein trafficking, protein labeling, protein visualization, surface-exposed protein determination, Reporter of Insertion and Surface Exposure (RISE)

## Introduction

Malaria disease kills > 400,000 people every year, and *Plasmodium falciparum* is the causative agent of this severe disease (World Health Organization, 2021). The *P. falciparum* has a complex life cycle involving sexual reproduction in a mosquito vector and asexual replication in the human host cells (Maier et al., 2019). The asexual stage is the most critical since patients start showing clinical symptoms at this stage, such as fever, jaundice, severe anemia, renal failure, and cerebral malaria, leading to death (Miller et al., 2002). These symptoms are triggered by the erythrocyte rupture during asexual multiplication. Within the erythrocyte, the *P. falciparum* parasite goes through various stages: ring, trophozoite, and schizont. Finally, the mature schizont releases 16–32 daughter merozoites after erythrocyte rupturing into the blood circulation to invade new erythrocytes (Bannister et al., 2000).

Although the blood-stage parasites account for the clinical manifestations of malaria, the erythrocyte environment is not suitable for parasite growth and asexual replication due to limited nutrients, inhospitable ionic composition, or acidic pH. To overcome these constraints, the effector proteins are exported by the malaria parasite into the host cell cytoplasm and membrane surface (Maier et al., 2009; Mundwiler-Pachlatko and Beck, 2013; Spillman et al., 2015; Sherling and van Ooij, 2016). With these effector proteins, the parasite alters the host cell physiology to fit its growth, extends its communication to the extracellular environment, and facilitates its immune evasion. The surface-exposed effector proteins (SEPs) attracted a huge amount of attention based on their functions and potential as drug targets. For example, the *P. falciparum* erythrocyte membrane protein 1 (PfEMP1) is well known for its crucial role in cytoadherence, mediating parasite virulence (Peters et al., 2007; Zhang et al., 2011; McMillan et al., 2013; Lee et al., 2019); the subtelomeric variable open reading frame proteins (STEVORs) and the repetitive interspersed family proteins (RIFINs) facilitate the adhesion to the endothelial lining of capillaries, evasion from splenic filtration, and possibly in antigenic variation (Wahlgren et al., 2017); and the cytoadherence-linked asexual gene proteins (CLAGs) contribute to the plasmodial surface anion channel (PSAC), which is functional for nutrient uptake and membrane permeability (Nguitragool et al., 2011; Desai, 2014).

However, the identification and determination of SEPs are still not straightforward, even after their importance has been recognized and confirmed for decades. The main challenges of SEP determination are due to its complicated trafficking procedure and limited labeling techniques. Here, the author briefly describes a well-accepted trafficking hypothesis of SEPs and summarizes the most prevalent and recent labeling techniques for SEP visualization and determination. These labeling techniques provide a brief introduction to SEP trafficking and highlight advanced quantitative and qualitative strategies in SEP studies.

## Delivery of Surface-Exposed Protein

The complexity associated with the SEP delivery from parasite cytoplasm to the erythrocyte membrane leads to a poor understanding of the trafficking mechanism. The SEPs must pass through at least two membranes before reaching their destination: the parasitophorous vacuole membrane (PVM) and the erythrocyte membrane. Hence, as the mature erythrocyte lacks trafficking machinery proteins, the parasite develops a novel trafficking system that includes structures with unknown functions, such as Maurer’s cleft and tubulovesicular network (TVN) (Spycher et al., 2006; Mundwiler-Pachlatko and Beck, 2013). Many reviews have reported possible trafficking procedures involving several steps. Firstly, the SEPs are carried and transferred into the parasitophorous vacuole (PV) by apical organelles during an invasion, from where they may stay until the late parasite stage. In the second step, these proteins are trafficked to the erythrocyte cytoplasm and cross the PVM by the *Plasmodium* Translocon of Exported proteins (PTEX), which is a PVM-located translocon. In the last step, before reaching the erythrocyte membrane, some proteins localize transiently to Maurer’s clefts, which is a parasite-derived membrane structure and considered an intermediate compartment in the transport or sorting of proteins (Maier et al., 2009; Mohandas and An, 2012; Mundwiler-Pachlatko and Beck, 2013; Spillman et al., 2015; de Koning-Ward et al., 2016; Neveu and Lavazec, 2019; Beck and Ho, 2021). This three-step trafficking procedure is a very much simplified hypothesis based on membranes. The actual trafficking process takes several complicated steps, making the visualization and determination of SEPs more challenging.

## Surface-Exposed Protein Labeling Strategies for Visualization and Determination

**Figure 1:**
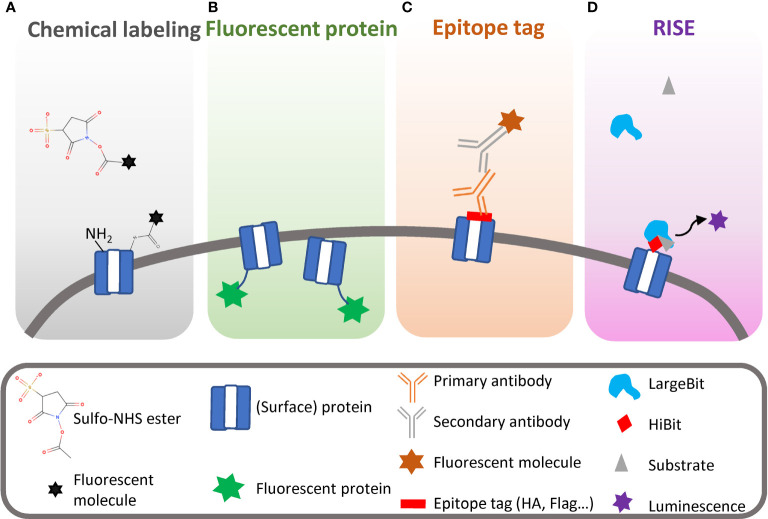
Different strategies to label surface-exposed proteins (SEPs) for visualization and determination. **(A)** Chemical labeling of SEPs by sulfo-NHS ester and forming covalent bond with an amine group. **(B)** Fluorescent protein as a reporter attached to target protein, showing both surface-exposed and peripheral proteins. **(C)** Bioaffinity-based strategy to detect surface-exposed protein. **(D)** RISE-specific detection of surface-exposed proteins using split luciferase.

Another major hurdle is that there is no effective labeling technique to visualize SEP trafficking and for confirming protein surface exposure. Until now, there are many different types of microscopes, such as fluorescence microscopes, confocal microscopes, electron microscopes, and the newly developed luminescence microscopes, to visualize protein locations in cells (De Niz et al., 2017). No matter which microscope is employed, the SEP labeling strategies are mainly classified into four theories depicted in **Figure 1**.

### Chemically Labeling Surface-Exposed Protein

Crosslinking is one of the simplest and most common techniques used for protein labeling. Here, the author used *N*-hydroxysuccinimide (NHS) ester as an example to elucidate the strategy. This technique uses the amine group from the N-terminus of each protein and the side chain of lysine (Lys, K) residues exposed on the cell surface to form a covalent bond with a crosslinker labeled with a fluorescent molecule (**Figure 1A**). As the crosslinker is labeled with a sulfo group which prevents it from permeating the cell membrane, only cell SEPs are labeled. The reactive group can be extended from the primary amine group to sulfhydryl or hydroxyl groups and even to photoreactive groups, allowing the chemical reaction to occur in various solutions for a certain purpose (Staros et al., 1986; Mattson et al., 1993). It is common to see a chemical labeling technique combined with mass spectrometry to identify SEPs. For example, Swearingen et al. isolated several surface-exposed proteins on sporozoites using a chemical labeling technique and identified the circumsporozoite protein (CSP) and the thrombospondin-related anonymous protein/sporozoite surface protein 2 (TRAP) *via* mass spectrometry (Swearingen et al., 2016). However, crosslinking has some drawbacks, especially for specific protein studies. This amine reaction can happen to any protein that has amine groups exposed on the cell surface, which results in a complicated protein profile revealed by this nonspecific labeling technique (Cohn et al., 2003; Florens et al., 2004). Also, occasionally, the reaction efficiency is dependent on certain pH and buffering compositions that are different from the physiological condition.

### Fluorescent Protein Labeling of Surface-Exposed Protein

To date, many different fluorescent proteins have been generated with various properties. The fluorescent proteins have played a pivotal role in understanding SEP trafficking in malaria (Wickham et al., 2001; Knuepfer et al., 2005; Ahmad et al., 2020). The Cowman group has extensively used fluorescent proteins as reporters to study SEP trafficking (Wickham et al., 2001; Knuepfer et al., 2005; Spycher et al., 2006; Rug et al., 2014). For example, they fused green fluorescent protein (GFP) with knob-associated histidine-rich protein (KAHRP), a key component of the knob structure on the infected erythrocyte membrane, and they defined the KAHRP trafficking route and binding domains by tracking the GFP signal (Wickham et al., 2001). However, this great reporter also has some limitations. The brightness of GFP is relatively low, especially for moving proteins, and the high background of GFP is not negligible for static proteins. The large size of the fluorescent proteins makes them difficult to express as it could affect the target protein features and adversely influence protein trafficking. Photobleaching can often happen to fluorescent proteins as well. The main restriction of applying fluorescent protein to SEP determination is that fluorescent protein cannot tell if the target protein is attached to a membrane-bound protein or inserted into the cell membrane, as shown in **Figure 1B**.

### Bioaffinity Strategy Labeling for Surface-Exposed Protein

Over the past decades, bioaffinity-based approaches for directly detecting SEPs have been developed and widely used on confocal microscopies, such as epitope tags (**Figure 1C**) (Knuepfer et al., 2005; Spycher et al., 2008; Rug et al., 2014; Ito et al., 2017; Gupta et al., 2020). Epitope tags can overcome some limitations that fluorescent protein suffers as a reporter. First, they are very small, which gives them more freedom to be inserted into the middle of the target protein and reduces the chance of affecting target protein features and trafficking. Second, the epitope can be a small sequence of a target protein where a specific primary antibody binds. Third, the secondary antibody can exponentially amplify the signal at a certain wavelength, which allows colocalization study for multiple proteins at once. Knuepfer et al. (2005) first used this technique to detect the surface-exposed N-terminus of KAHRP protein inside live cells and confirmed PfEMP1 is surface exposed combined with GFP labeling.

Similarly, the epitope tag technique also has its limitations. This strategy is a bioaffinity-based approach dependent on antibody–antigen specificity and affinity. The specificity and affinity are different for each antibody–antigen pair, leading to incorrect interpretations if wrong antigen binding happens. Epitope-based confocal can define the protein localization but cannot distinguish SEPs from peripheral proteins due to limited resolution. Radiation damage is another concern since phototoxicity and photobleaching often appear on fluorescent molecules, which impedes kinetic data collection.

### RISE Visualizes and Determines Surface-Exposed Protein

Recently, we have developed an approach called Reporter of Insertion and Surface Exposure (RISE), which can specifically detect SEPs (**Figure 1D**) (Shao et al., 2022). The basic of the RISE approach is a split luciferase that can oxidize the substrate and generate luminescence as a signal. The split luciferase consisted of two complementary nonenzymatic fragments known as HiBit (1.3 kDa) and LargeBit (18 kDa) with a high affinity of 700 pM (Azevedo et al., 2014; Dixon et al., 2016). We applied the 11 aa HiBit on an extracellular loop of CLAG3 protein that is the key determent of PSAC (Nguitragool et al., 2011; Schureck et al., 2021; Shao et al., 2022). Upon the addition of extracellular LargeBit, a luminescence signal is detected because of the completion of the fully functional split luciferase. With this approach, we tracked CLAG3 trafficking and surface exposure through the entire parasite asexual cycle (Shao et al., 2022). Furthermore, we also performed single-cell analysis and quantified luminescence intensity for each parasite under luminescence microscope imaging (Shao et al., 2022). Since this approach is an enzymatic activity-based platform, it can easily exclude a false-positive interpretation. Along with this, RISE can especially distinguish SEPs from peripheral proteins because only SEPs have luminescence production. However, this approach requires a specific luminescence microscope for a single-cell-level analysis, and the target surface protein must tolerate the HiBit insertion.

## Conclusions and Prospects

In this opinion, the author compared and summarized four different labeling strategies to determine and visualize SEPs on *P. falciparum*-infected erythrocytes. Parasites export SEPs to remodel the erythrocyte membrane to accomplish their growth, pathogenesis, and immune evasion. However, the delivery and determination of these SEPs is a mystery due to the complicated trafficking procedure, the involvement of novel structures derived from parasites, and limited techniques. The strategies listed in this opinion have their pros and cons, but, RISE has all the advantages that an epitope tag technique could provide and overcomes the issue of distinguishing SEPs from periphery proteins faced in the other strategies. Furthermore, RISE can provide quantitative and kinetic data *via* luminescence intensity analysis. With the strong signal intensity, high sensitivity, super selectivity, and specificity of split luciferase, we envisioned that RISE could be a beneficial technique to explore SEPs from other pathogen-infected cells or even cancerous cells.

## Author Contributions

The author confirms being the sole contributor of this work and has approved it for publication.

## Funding

JS is supported by the Intramural Research Program of the National Institutes of Health, National Institute of Allergy and Infectious Diseases.

## Conflict of Interest

The author declares that the research was conducted in the absence of any commercial or financial relationships that could be construed as a potential conflict of interest.

## Publisher’s Note

All claims expressed in this article are solely those of the authors and do not necessarily represent those of their affiliated organizations, or those of the publisher, the editors and the reviewers. Any product that may be evaluated in this article, or claim that may be made by its manufacturer, is not guaranteed or endorsed by the publisher.
